# CYP1A1 Induction in the Colon by Serum: Involvement of the PPARα Pathway and Evidence for a New Specific Human PPREα Site

**DOI:** 10.1371/journal.pone.0014629

**Published:** 2011-01-31

**Authors:** Pierre-Henri Villard, Fabrice Barlesi, Martine Armand, Thi-Mai-Anh Dao, Jean-Marc Pascussi, Francis Fouchier, Serge Champion, Claire Dufour, Christian Giniès, Ayman Khalil, Marie-Josephe Amiot, Yves Barra, Eric Seree

**Affiliations:** 1 INRA, UMR1260, Nutriments Lipidiques et Prévention des Maladies Métaboliques, Marseille, France; 2 Faculté de Médecine, Université de la Méditerranée Aix-Marseille 2, IPHM-IFR 125, Marseille, France; 3 Thoracic Oncology, Université de la Méditerranée – Assistance Publique Hôpitaux de Marseille, Marseille, France; 4 Institut de Recherche pour le Développement, UMR 5096 (CNRS-IRD-Université Perpignan), Montpellier, France; 5 UMR 408 Safety and Quality of Plant Products, INRA, University of Avignon, Site Agroparc, Avignon, France; Dr. Margarete Fischer-Bosch Institute of Clinical Pharmacology, Germany

## Abstract

**Background:**

We previously showed that blood serum induced cytochrome P450 1A1 (CYP1A1) monooxygenase expression in vitro.

**Objective:**

Our purpose was (i) to identify the molecular mechanism involved and (ii) to characterize the inducer compound(s) in serum involved at least in part.

**Methods:**

Serum was fractionated on hydrophobic columns. PPARα involvement was demonstrated by gene reporter assays, DNA mutagenesis and EMSA. Gene expression was evaluated by qRT-PCR. Serum samples were analyzed using HS-SPME-GC-MS.

**Results:**

The inductive effect of serum did not depend on the AhR pathway and was enhanced by cotransfection of PPARα cDNA. Mutations in the PPAR response elements of the CYP1A1 gene promoter suppressed this effect. One of the PPRE sites appeared highly specific for human PPARα, an unreported PPRE property. A link was found between CYP1A1 inducibility and serum hydrophobic compounds. Characterization of sera showed that hexanal, a metabolite produced by peroxidation of linoleic acid, was involved in CYP1A1 induction by serum, possibly along with other serum entities.

**Conclusion:**

We demonstrate that serum induces CYP1A1 via the PPARα pathway and that hexanal is one of the serum inducers. The two PPRE sites within the CYP1A1 promoter are functional and one of them is specific for PPARα.

## Introduction

Cytochromes P450 (CYP) are monooxygenases involved in the metabolism and degradation of xenobiotics, including procarcinogens such as arylamines and polycyclic aromatic hydrocarbons (PAHs) [1]. Among the CYP, CYP1A1 plays a physiological role in the degradation of estradiol into 2-OH-estradiol [2]. These metabolisms elicit the production of reactive oxygen species (ROS). CYP1A1 gene expression is mainly regulated by the aryl hydrocarbon receptor (AhR) activated by xenobiotics including dioxins and polycyclic aromatic hydrocarbons [3]. Endogenous agonist AhR ligands (such as bilirubin, tryptophan-*N*-formylated derivatives and lipoxin A4) have already been identified, together with an endogenous antagonist, 7-ketocholesterol [4], [5], [6], [7]. However, the role of endogenous AhR ligands in cell physiology remains poorly understood. The activated AhR migrates into the nucleus, interacts with its partner, the aryl hydrocarbon receptor nuclear translocator (ARNT) and the heterodimer binds DNA at specific dioxin-responsive elements (DRE) [8].

There is little data available on CYP1A1 expression stimulation by other regulatory pathways. Retinoic acid (RA) exerts a weak transactivation through a RARE (retinoid acid responsive element) sequence in the CYP1A1 promoter [9], but essentially inhibits AhR activity through SMRT displacement [10], [11]. We previously identified PPARα as a mediator of CYP1A1 induction [12]. Free fatty acids (FA) may act as PPAR ligands. Polyunsaturated FA (PUFA) are PPARγ and PPAPα agonists [13], [14], but less is known about saturated, peroxidized, halogenated or thio-derivatives of FA. We hypothesized that serum containing a high level of FA could induce CYP1A1 expression through PPARα activation by one or more discrete FA species or derivatives.

Several clinical reports have shown that local CYP1A1 overexpression correlates with predisposition to various human cancers, including colon and non-small cell lung cancers (NSCLC) [15].

We report here that serum-mediated CYP1A1 induction involves PPARα and two PPRE sites within the CYP1A1 promoter (positions –931/-919 and –531/-519, named PPRE1 and PPRE2), and that the proximal PPRE site is a new human PPARα-specific consensus site. In parallel, we have characterized one serum product derived from the peroxidation of linoleic acid, hexanal, that is at least partly responsible for intestinal CYP1A1 induction.

## Materials and Methods

### Culture and cell treatments

Human colic adenocarcinoma cells CaCo-2 and HT29-D4, human hepatoma cells Hep G2, human pulmonary cells A549 and primary human keratinocytes (obtained under ethical conditions), were cultured as previously described [16]. After 80% confluence, cells were starved for 24 h without FBS (replaced by 0.2% BSA) and treated for 6 h with 20% FBS or a low-FA FBS (FBSLess); or 1 µM of 3-methyl-cholanthrene (3-MC); or 100 µM of WY-14643; or 1 µM of retinoic acid (RA); or 200 µM of free FA (palmitic, stearic, linoleic, α-linolenic, γ-linolenic, arachidonic or eicosapentaenoic acid); or 5, 10, 25, 50, 100 and 200 µM of hexanal (Sigma, France). Control cells were treated with the solvent used to dissolve the compounds (DMSO).

### Quantitative RT-PCR experiments

Total RNA was isolated using a Nucleospin RNAII kit (Macherey-Nagel, France) and reverse-transcribed at 37°C for 1 h using GibcoBRL M-MLV reverse-transcriptase (Life Technologies, France) and random primers. CYP1A1 mRNA expression, normalized to β2-microglobulin, was determined using the LightCycler System (Roche Diagnostics, France). The CYP1A1 and β2microglobulin primers used were:

CYP1A1-S: 5′AAGAGGAGCTAGACACAGT3′

CYP1A1-AS: 5′GAAACCGTTCAGGTAGGA3′

β 2m-S: 5′CCGACATTGAAGTTGACTTAC3′

β 2m-AS: 5′ATCTTCAAACCTCCATGATG3′

PCR was performed as previously described [17]. The results are expressed as relative expression levels (REL). At least three independent experiments were carried out in triplicate.

### CAT assays

Cells were placed in six-well plates and transfected using lipofectin (Life Technologies, France) with 1 µg of a chimeric construction including the −1140/+80 region of CYP1A1 gene, (pRNH25c), or 1 µg of DRE-TK-CAT construct [16] driving chloramphenicol acetyltransferase (CAT). In some experiments, 1 µg of psG5 PPARαcDNA [18] was cotransfected. After an 18 h incubation period, cells were treated 24 h later with 20% FBS or 1 µM 3-MC or 100 µM WY-14643 and harvested after a further 24 h. CAT expression was then evaluated by quantifying CAT protein using the CAT Elisa System (Roche Diagnostics, France) [19]. The transfection efficiency was normalized using beta-Galactosidase expression vector. At least three independent experiments were carried out in triplicate.

### LUCIFERASE assays

Cells were placed in six-well plates and transfected using lipofectin with 1 µg of a chimeric construction including three PPRE sequences driving luciferase (LUC) gene expression (PPRE-TK-LUC), and treated as described above. LUC enzymatic activity was then evaluated. The transfection efficiency was normalized using beta-Galactosidase expression vector. At least three independent experiments were carried out in triplicate.

### Site-directed mutagenesis of CYP1A1 promoter

PPRE mutations of pRNH25c were introduced using the QuickChange site-directed mutagenesis kit (Stratagene, France) to obtain pRNH25c-ΔPPRE as previously described [12]. Cells were transfected by the pRNH25c or the pRNH25c- ΔPPRE and after a 48 h treatment with 100 µM WY-14643, 20% FBS or 1 µM 3-MC the CAT expression was evaluated as described above. The transfection efficiency was normalized using beta-Galactosidase expression vector.

### FBS treatment by XAD2 column or active charcoal

50 ml of FBS was run through either an XAD2 column (Sigma, France) or active charcoal. The retained hydrophobic elements were eluted from the XAD2 column with methanol, and from active charcoal with methanol, ethyl acetate or hexane. After solvent evaporation the eluted compounds were resuspended in 50 ml of FBS free culture medium.

### *In vitro* translation and electromobility shift assays (EMSA)

EMSA were performed using PPARα and RXRα prepared by *in vitro* translation (Promega, France). Proteins were incubated for 20 minutes at room temperature with 50.000 cpm of T4 polynucleotide kinase-labeled oligonucleotides (in 10 mM Tris pH 8.0, 100 mM KCl, 10% glycerol, 1 mM dithiothreitol, 1 µg of poly-dIdC and 0.5 µg of salmon sperm) and separated on a 4% polyacrylamide gel. The oligonucleotides used as either radiolabeled probes or competitors (sense strands are shown, with core sequence underlined and mutation in bold-face) as previously described [12].

Autoradiography was carried out on Kodak X-AR film.

### Determination of lipid serum composition

Total lipids were extracted from 1 ml aliquots of FBS and FBSLess using the Folch method modified by Hernell [20], [21]. The chloroform fraction containing lipids was evaporated to dryness under nitrogen, and the lipid pellet was suspended in isopropanol. Triglycerides, total cholesterol and free fatty acids were assayed by colorimetry using specific commercial kits (Triglyceride/Free glycerol reagent from Sigma, Cholesterol CHOD-PAP from Roche Diagnostic, NEFA from Randox, respectively), and phospholipids were assayed by phosphorus determination [22]. Fatty acid profiles were determined after methylation with BF_3_-methanol (Sigma, St Louis, MO) by gas chromatography [23] using a Perkin Elmer Autosystem XL (Perkin Elmer, Courtaboeuf, France) equipped with a fused silica capillary column (Omegawax 250, 30 m ×0.25 mm i.d., film thickness 0.25 µm; Sigma-Supelco), equipped with a flame ionization detector and the Turbochrom software. Hydrogen was used as carrier gas. The oven temperature was ramped from 60°C to 215°C at 45°C/min. Fatty acids were identified by their retention times against standards (PUFA 2, Sigma-Supelco).

### HS-SPME-GC-MS analysis of volatile compounds in FBS and FBSLess sera

Substances derived from FA peroxidation were sought in both sera. Headspace solid-phase microextraction (HS-SPME) was used to extract the volatile organic compounds, which then underwent qualitative analysis by gas chromatography–mass spectrometry (GC-MS). HS-SPME was performed with an AOC-5000 autosampler: 1 ml of serum was placed in a sealed 20 ml vial and thermostated at 37°C for 15 min before introduction of the fiber (carboxenTM- PDMS, length 10 mm, thickness 75 µm, Supelco Inc., Bellefonte, USA). The fiber was exposed to the vapor phase for 5 min to sample the volatile compounds, and then inserted into the injection port of the GC-MS (GC QP 20120, Shimadzu) using an inlet liner (0.75 mm i.d.) set at 250°C for thermal desorption for 3 min. (splitless mode, purge opened after 0.5 min.). Volatiles were separated on an UBWAX column (30 m ×0.25 mm, 0.5 µm, Interchim, Montluçon, France). The carrier gas was He at a velocity of 35 cm.s^−1^. The oven program temperature was as follows: 40°C for 1 min, 3°C min^−1^ to 100°C and 5°C min^−1^ to 220°C. The mass spectrometer was operated in the electron impact mode at 70 eV in the *m/z* range 29–300 at a speed of 2 scans s^−1^. The temperatures of the ion source and transfer line were 200°C and 240°C. Detected compounds were identified by matching spectra against the NIST library (v.2.0).

### Statistical Analysis

Results are expressed as means ± SD. Data were analyzed using Wicoxon signed rank test. *P* values less than 0.05 were considered significant. All analyses were done using the STATVIEW software (Abacus Concepts, Berkeley, CA, USA).

## Results

### FBS induced CYP1A1 in different cell lines independently of AhR

**Figure 1:**
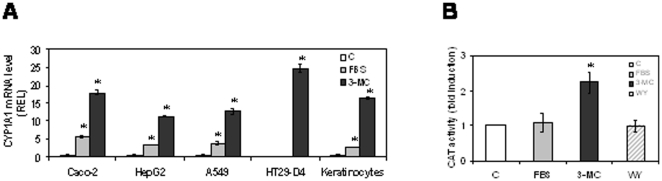
CYP1A1 induction by FBS is independent of AhR. 1A: CYP1A1 mRNA levels in various cell lines after a 6 h treatment by 20% FBS or 1 µM 3-MC. 1B: CaCo-2 cells were transfected with the DRE-TK-CAT construct and treated with 20% FBS, 1 µM 3-MC or 100 µM WY-14643. CAT expression was evaluated 24 h later.

We studied the inducibility of CYP1A1 mRNA by serum in A549 CaCo-2, HepG2, HT29-D4 cells and human primary keratinocytes. As shown in Figure 1A, 3-MC induced CYP1A1 mRNA in all the cell lines tested, while serum induced CYP1A1 expression in all the cell lines except the human HT29-D4 adenocarcinoma cell line. As CaCo-2 cells are high-responsive, we chose this cell line to study the molecular mechanism involved in the CYP1A1 induction process by serum. We also performed reporter gene assays where CAT expression was driven by two DRE sequences. As shown in Figure 1B, in CaCo-2 cells, this construct was inducible by 3-MC but not by serum. In addition we studied, in HT29-D4 and CaCo-2 cells, the AhR protein nuclear translocation after 3-MC or FBS treatments. Result (data not shown) shown that 3-MC, but not serum, is able to induce AhR nuclear translocation in the two studied cell lines.

### CYP1A1 inducers present in serum are hydrophobic

**Figure 2:**
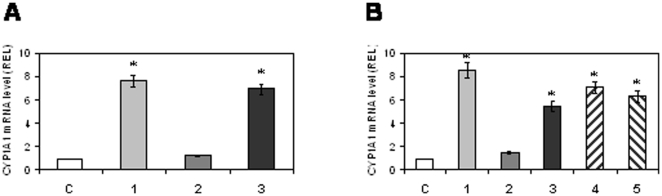
Effect of FBS fractions on CYP1A1 mRNA levels. 2A: Results obtained after XAD2-bound or unbound fractions of FBS. C: control cells; 1: cells treated with FBS; 2: cells treated with XAD2-unbound fraction; 3: cells treated with XAD2-bound fraction eluted with methanol. 2B: Results obtained after charcoal-bound or unbound fractions of FBS. C: control cells; 1: cells treated with FBS. Cells were treated with either unbound-charcoal fraction (lane 2) or compounds eluted from charcoal by methanol (lane 3), ethyl acetate (lane 4) or hexane (lane 5).

We fractionated FBS by chromatography through XAD2 or active charcoal columns to study the chemical nature of the CYP1A1 inducers. Retained hydrophobic compounds were eluted from the XAD2 column by methanol and from the active charcoal by methanol, ethyl acetate, or hexane. As shown in Figure 2A and 2B, CYP1A1 inducers contained in serum were retained by both XAD2 and charcoal, since there was no induction with the flow-through fraction (Figure 2A, lane 2 and Figure 2B, lane 2). The inducers were eluted from both XAD2 (with methanol) (Figure 2A, lane 3) and charcoal (with ethyl acetate, hexane, or methanol), as the eluate elicited CYP1A1 induction (Figure 2B, lanes 3, 4, 5). Hence the serum inducing compound(s) was more likely a hydrophobic compound (Figure 2) rather than proteins, as MgSO4 protein precipitation had no effect on CYP1A1 induction by FBS (data not shown).

### CYP1A1 induction by FBS involves PPARα

**Figure 3:**
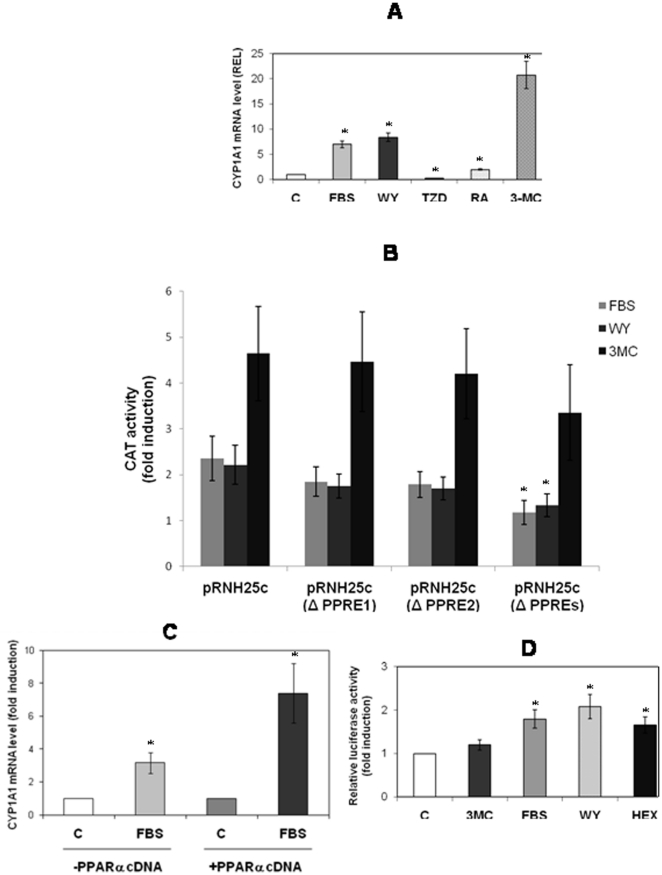
Effect of PPRE on CYP1A1 promoter activation. CaCo-2 cells were either left untreated (C) or treated for 6 h with either: 20% FBS, 100 µM WY-14643, 200 µM 2, 4-thiazolidinedione, 1 µM RA, or 1 µM 3-MC (3A). Before treatments CaCo2 cells were transfected with wild-type pRNH25c or pRNH25c-ΔPPRE1, pRNH25c- ΔPPRE2 or pRNH25c- ΔPPREs (3B) and treated with serum or 100 µM WY-14643 or 1 µM 3-MC. CAT expression was evaluated. Statistical analyses were performed by comparing mutated PPRE constructions to the wild-type construct. 3C: Effect of PPARα cDNA transfection on CYP1A1 mRNA induction by FBS. 3D: CaCo-2 cells were transfected by PPRE-TK-LUC and treated with 1 µM 3-MC, 20% FBS, 100 µM WY-14643 or 25 µM hexanal (HEX). C: cells treated with solvent.

In addition to the AhR-dependent DRE sites, CYP1A1 can also be induced through two PPRE sites and one RARE. We treated CaCo-2 cells for 6 h with FBS, WY-14643 (a PPARα ligand), TZD (a PPARγ ligand), RA or 3-MC. As shown in Figure 3A, TZD did not increase CYP1A1 mRNA levels, whereas there was a marked induction with the WY-14643, FBS and 3-MC treatments and a weaker induction with RA.

To confirm that FBS was able to induce CYP1A1 through PPRE sequences, we transfected CaCo-2 cells with either pRNH25c, or pRNH25c- ΔPPRE. Cells were treated with FBS or WY-14643 for 48 h. As shown in Figure 3B, FBS and WY-14643 efficiently induced CYP1A1 promoter transactivation (2.5-fold) while the mutation of one PPRE site alone slightly reduced luciferase activity, and the mutations of the two PPRE sites abrogated it. We observed that CYP1A1 promotor activation by 3-MC is independent of PPRE sites, this activation being lower when PPRE sites were mutated.

FBS-mediated CYP1A1 induction was increased by cotransfection of a PPARα expression vector (2-fold) (Figure 3C). Finally, as shown in Figure 3D, both FBS and WY-14643 were able to transactivate reporter constructs driven by a canonical PPRE sequence, while no induction was observed with 3-MC. In addition, WY-14643 was unable to transactivate DRE sequences (Figure 1B).

**Figure 4:**
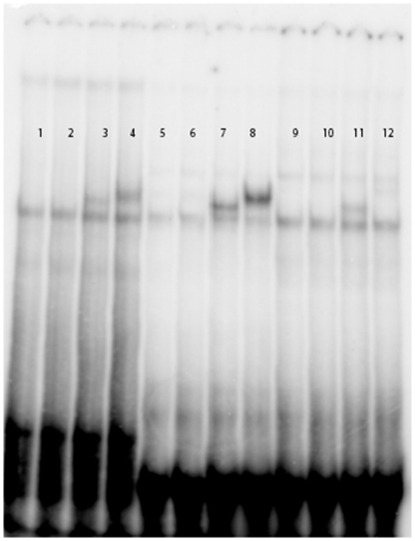
Detection of PPARα binding on the two CYP1A1 PPRE sites by Gel shift assay. A PPRE canonical sequence, the CYP1A1 PPRE1 (position –931/-919) and the CYP1A1 PPRE2 (position –519/-531) were tested. 1: TNT + PPRE canonical sequence; 2: TNT + RXR + PPRE canonical sequence; 3: TNT + RXR + PPARα + PPRE canonical sequence; 4: TNT + RXR + PPARγ + PPRE canonical sequence; 5: TNT + PPRE 1; 6: TNT + RXR + PPRE 1; 7: TNT + RXR + PPARα + PPRE 1; 8: TNT + RXR + PPARγ + PPRE 1; 9: TNT + PPRE2; 10: TNT + RXR + PPRE2; 11: TNT + RXR + PPARα + PPRE2; 12: TNT + RXR + PPARγ + PPRE2.

We investigated the PPARα-binding ability of each CYP1A1 PPRE. Given that TZD did not induce CYP1A1 expression and that CaCo-2 cells express PPARα and PPARγ, we hypothesized that these two PPRE sequences might act through PPARα. This hypothesis was supported by EMSA. As shown in Figure 4, we compared the specificity of each CYP1A1 PPRE sequence toward PPARα or PPARγ. The results (Figure 4, lanes 11–12) indicate that the PPRE2 core is specific for PPARα. PPARγ was unable to recognize the PPRE2 core (Figure 4, lane 12). By contrast, PPARγ recognized a PPRE core sequence (Figure 4, lane 4) as well as PPRE1 (Figure 4 lane 8).

### Characterization of the serum compound involved in CP1A1 induction

**Figure 5:**
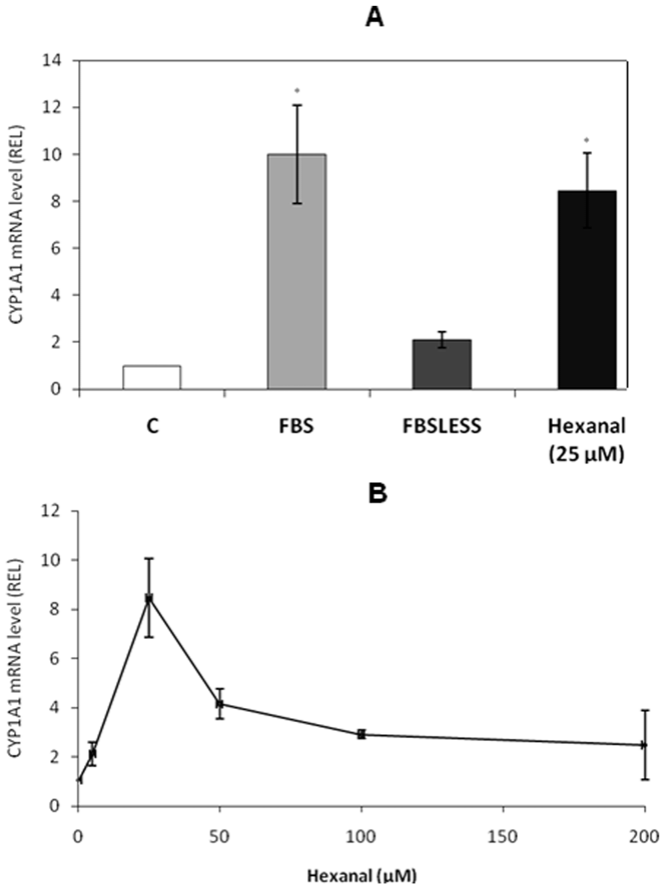
Effect of FBS, FBSLess (FBS with low fatty acids levels) and hexanal on CYP1A1 mRNA levels. C: control cells; FBS: cells treated with 20% FBS; FBSLess: cells treated with 20% FBSLess; hexanal: cells treated with 25 µM hexanal (5A). Effect of various hexanal concentrations (5, 10, 25, 50, 100 and 200 µM) on CYP1A1 mRNA level (5B).

**Figure 6:**
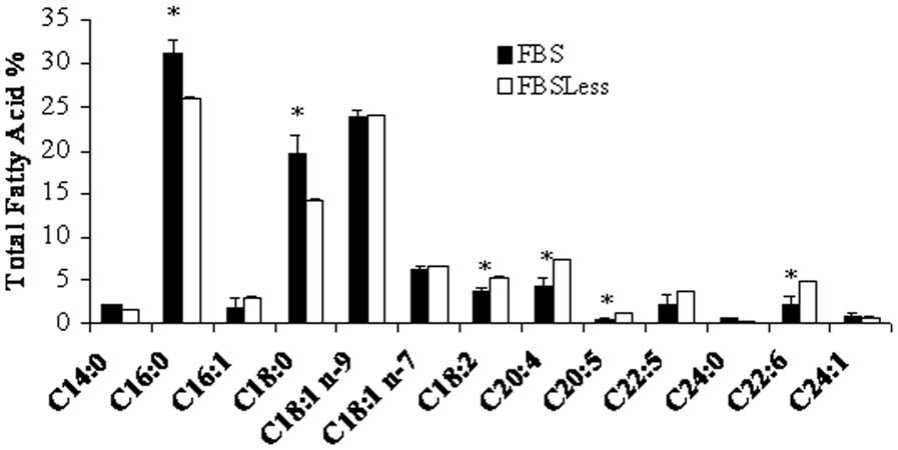
Fatty acid profile of total lipid extract of FBS (black bars) and FBSless (white bars). The data are expressed as weight % of total fatty acids and represent the mean ± SEM of three different determinations. *indicates significant differences between the sera for a given fatty acid (*p*<0.05).

**Figure 7:**
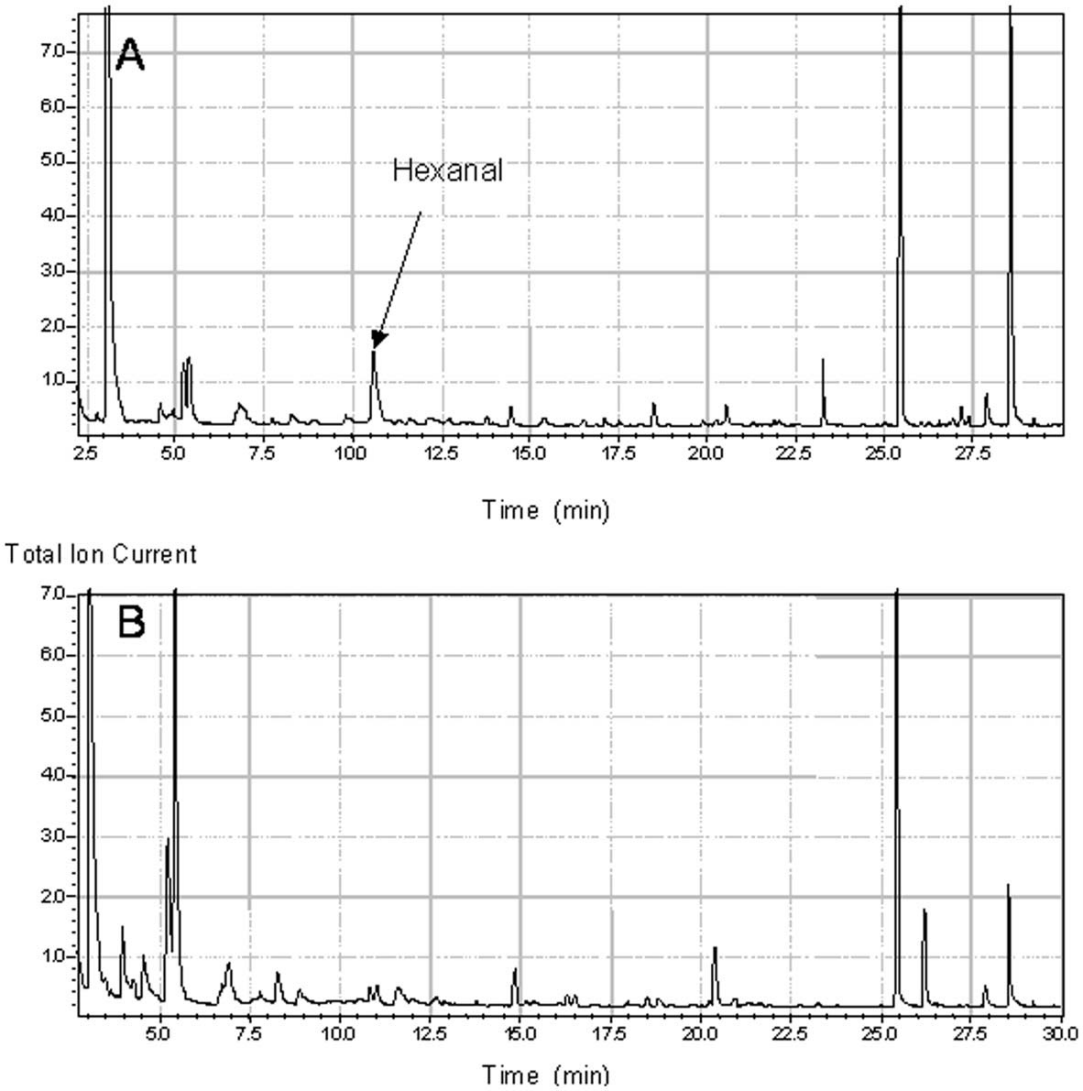
HS-SPME-GC-MS analyses of the volatiles in FBS (A) and FBSLess (B) sera. Analyses were conducted with a 1 mL aliquot of each serum. The black arrow indicates the presence of hexanal. List of the different substances found in the sera in order of retention time: for A, acetone, 2-butanone, 2-propanol, ethanol + CH2CL2, hexanal, 1-butanol, 1-penten-3-ol, pentanol, 1-hexanol or isomer, cyclohexanol, acetic acid, 1-hexanol, 2-ethyl; for B, hydrocarb, hydrocarb C8 # 1, hydrocarb C8 # 2, hydrocarb C8 acetone, 2,4-dimethyl-1-heptene, 2-butanone, 2-propanol, ethanol, unknown, styrene, cyclohexanone, cyclohexanol, *m*-di-tert-butylbenzene, acetic acid, 1-hexanol, 2-ethyl.

Hydrophobic entities present in FBS (Figure 2) induce CYP1A1 through a PPARα pathway (Figure 3). To determine their nature we analyzed two sets of serum harboring different CYP1A1 induction capabilities. The results, presented in Figure 5A, demonstrate that the serum usually used in this study (FBS) induced CYP1A1, unlike the second serum characterized by a naturally low FA content (FBSLess). We analyzed the lipid composition (Table 1) and FA profile (Figure 6) of each serum. The results show that FBS had higher lipid content and contained four times more free FA than FBSLess (Table 1). In addition, FBS contained a higher proportion of saturated FA (54% *vs.* 42%), and a lower proportion of PUFA (13% *vs.* 23%) than did FBSLess, but the same proportions of mono-unsaturated FA (33% *vs.* 34%) (Figure 6). These data suggest a link between the free FA level and the inducibility of CYP1A1. In further experiments using cells treated with various free FA (palmitic acid, stearic acid, linoleic acid, linolenic acid, arachidonic acid and eicosapentaenoic acid) we did not observe any CYP1A1 induction (data not shown). As PUFA levels were lower in FBS than in FBSLess, and as PUFA are very sensitive to peroxidation, we performed HS-SPME-GC-MS analysis of the two batches of serum to look for oxidative derivatives (Figure 7A and B). We found that in contrast to FBSLess (Figure 7B), FBS was rich in hexanal (approx. 28 µM), a substance arising specifically from the peroxidation of linoleic acid (Figure 7A). Among the different entities found and presented in Figure 7, the only major substance detected derived from FA peroxidation was hexanal. We therefore treated CaCo-2 cells with hexanal. The results, presented in Figure 5, show that hexanal induced CYP1A1 to an extent similar to that obtained using FBS (7.2- and 10-fold respectively). This finding confirmed the role of hexanal in CYP1A1 induction by serum. We realized a set of experiments to confirm the role of hexanal in CYP1A1 induction via PPARα. We treated (Figure 5A) CaCo-2 cells with 25 µM of hexanal and we showed that CYP1A1 was induced. We also studied the effect of different hexanal concentrations in CYP1A1 induction. The results presented in Figure 5B demonstrated that the CYP1A1 inductibility was linked to the hexanal concentration between 1 to 25 µM. Above 25 µM of hexanal, the CYP1A1 induction decrease strongly due to the toxicity of this aldehydic compound.

## Discussion

Although AhR was initially considered as mandatory for CYP1A1 gene expression [24], our data demonstrate that serum induces CYP1A1 expression and that this induction involved PPARα. Fractionation experiments on FBS suggested that hydrophobic entities were involved. These were probably not proteins, since ammonium sulfate precipitation of proteins did not affect CYP1A1 induction by FBS (data not shown). These findings led us to hypothesize that serum induced CYP1A1 via a ligand able to bind to a member of the steroid hormone nuclear receptor family.

The CYP1A1 promoter harbors several regulatory elements including two PPRE and one RARE. Our team had previously shown that the predominant effect of retinoids (RAR ligands) on CYP1A1 expression was an inhibition of AhR-mediated induction, acting through interference between the SMRT corepressor and AhR [10], [11]. This RARE sequence is thus irrelevant in the context of the present study since FBS potentiates AhR-mediated CYP1A1 induction [16].

CaCo-2 cells express a high level of PPARγ, but TZD failed to induce CYP1A1. By contrast, we demonstrated that PPARα played a significant role in CYP1A1 up-regulation. The involvement of PPRE sequences in the serum induction is shown in our study (Figure 3). We note that the PPRE2 site located at position–519/-531 is specific for the PPARα isotype and is not recognized by PPARγ (Figure 4). This is the first demonstration of a differential specificity among PPREα. PPARγ is able to bind to the PPRE1 site located at position –931/-919 (Figure 4), but the specific PPARγ ligand TZD was unable to induce CYP1A1 (Figure 3A), as previously described for troglitazone [12].

Mutagenesis of each PPRE site or of the two sites suppressed the FBS and WY induction but not the 3-MC-mediated CYP1A1 induction (Figure 3B). We observed a slight decrease of luciferase activity after a 3-MC treatment in the two PPRE mutation groups, but this decrease is not significant. Furthermore, we showed in Figure 1B that the PPARα ligands are not able to activate the XRE. We therefore suppose that the absence of PPARα binding on the CYP1A1 promoter after mutation can induce a conformational change of the promoter structure leading to a decrease of the activity of AhR on XRE sequences.

Subjects exposed to AhR agonists and exhibiting high blood levels of endogenous PPARα ligands would therefore be expected to present a greater risk of developing intestinal or pulmonary cancers and/or other diseases related to CYP1A1 overexpression. This is in agreement with epidemiological data showing that a high-fat diet increases the risk of colon cancer [25]. Dietary exposure to food-derived heterocyclic amine carcinogens and polycyclic aromatic hydrocarbons has been proposed as a specific risk factor [26], [27], [28].

Our results demonstrate that at least one of the serum inducing compounds is hexanal. This substance is one of the primary oxidative metabolites belonging to the saturated aldehyde family and is the one most abundantly formed during peroxidation of linoleic acid [29], [30], [31]. LA availability in humans is high. It is supplied in the Western diet, with a consumption in the range 8-12 g/d in adults [32], [33], [34], and is commonly present in the human body in blood, in the range 18–27% of total plasma fatty acids [34], [35], and in tissues. LA can undergo oxidative stress, leading in part to the generation of hydroxy radicals in a variety of pathological states. More specifically, hexanal has been proposed as a volatile cancer biomarker found in blood for lung cancer [36] and more recently for liver cancer [37]. However, nothing has yet been reported on the impact of hexanal on intestinal cell function. Here we describe a new impact of hexanal on a human intestinal cell line. People with high circulating hexanal blood levels, leading to a higher CYP1A1 expression, may thus be at higher risk of developing a colorectal cancer, as was demonstrated for genetic polymorphisms increasing CYP1A1 expression or activity [28].

In conclusion, we have characterized the mechanism involved in CYP1A1 induction in the human colon by serum and we show that hexanal may be at least partly responsible for this induction. PPARα transcription factor and two PPRE sites within the CYP1A1 promoter are involved, and one PPRE, the PPRE2 site, appears to be specific for the human PPARα.

**Table 1:** Lipid composition of FBS and FBSLess (mM) sera.

Lipids	FBS	FBSLess
Triglycerides, mM	1.26±0.02*	0.86±0.03
Total cholesterol, mM	1.11±0.03*	0.72±0.01
Free fatty acids, mM	0.47±0.02*	0.12±0.01
Phospholipids, mM	0.72±0.04*	0.23±0.01
Total lipids, g/L	1.8±0.03*	1.02±0.06

Measurements were made in triplicate. Data represent mean ± SEM.

*indicates significant differences between the sera for a given lipid parameter (Mann-Whitney *U* test, *p*<0.05).
